# Endodontic bioceramics: current and futurity aspects

**DOI:** 10.3389/froh.2025.1699547

**Published:** 2026-01-12

**Authors:** Roma M, Karthik Shetty, Laxmish Mallya, Krishna Prasad Shetty

**Affiliations:** 1 Department of Conservative Dentistry and Endodontics, Manipal College of Dental Sciences Mangalore, Manipal Academy of Higher Education, Manipal, India; 2 Department of Clinical Science, College of Dentistry, Centre of Medical and Bio-Allied Health Science Research, Ajman University, Al-Jruf/Ajman, United Arab Emirates

**Keywords:** bioceramics, vital pulp therapy, root canal therapy, endodontic microsurgery, regenerative endodontic treatment, health

## Abstract

Excellent bioactivity and biocompatibility make bioceramics a popular choice in dentistry, especially in endodontics. The most commonly utilised bioceramic in endodontics is mineral trioxide aggregate (MTA). Several emerging bioceramics showed promise for endodontic treatment. This study discusses bioceramics and their use in endodontic treatments like as root-end filling, root canal therapy, vital pulp therapy, apexification/regenerative endodontic treatment, perforation repair, and root defect repair. The applicable research from 1990 to 2023 was screened using keywords in PubMed and Web of Science. According to current research, MTA in the management of endodontic disease is well supported. New bioceramics including Biodentine, EndoSequence, and calcium-enriched mixes have demonstrated encouraging clinical results, but additional controlled trials must be conducted to establish their use in endodontics. To address endodontic problems, bioceramics must be improved for their biologic activity, including antibacterial activity, mechanical qualities, and reduced setting time and solubility. The purpose of this review is to provide an overview of the state of bioceramic technology and to investigate potential avenues for further investigation to improve its regenerative and therapeutic potential in endodontics. *Research Question: What developments and potential uses can enhance the endodontic bioceramic-based therapies' regeneration results?*

## 1 Introduction

**Figure 1:**
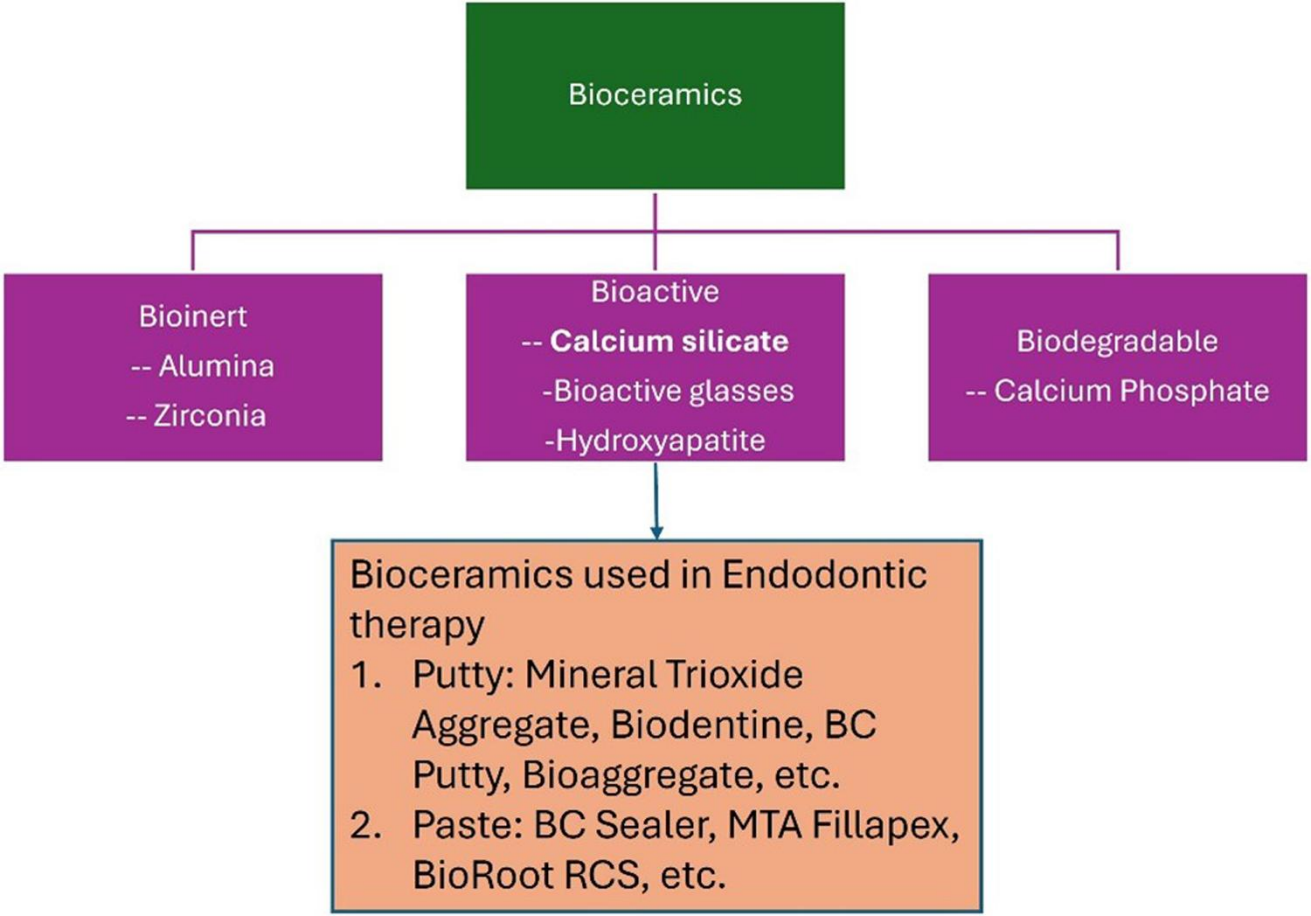
Classification of bioceramic materials.

In the early 1990s, bioceramics emerged as an emerging dental component in endodontics. The assessment of dental biomaterials identified bioceramics as an area of emphasis from 2007 to 2019 (1). Bioceramics are biomimetic and biocompatible compounds like bioactive glasses, hydroxyapatite, calcium phosphate, calcium silicate etc. “Bioceramics are categorised as biologically inert, bioactive, or biodegradable depending on their interaction with surrounding tissues,” as not all bioceramics share all three properties (2, 3). (Figure 1). Bioactive bioceramics, such as calcium silicate-based cements (CSCs), are commonly utilised in endodontics (4). Besides contributing to their mechanical and chemical features, CSCs are crucial in pulp space therapy or root canal therapy for their compatibility and biological activity (5).

In the past 30 years, there has emerged a major fascination in creating biologically active materials that can stimulate tissue repair. Mineral trioxide aggregate (MTA), being the initial bioceramic material in endodontics, has been extensively explored. MTA is a Portland cement-based material with superior biological compatibility and hermetic properties. In 1993, it was launched in dentistry as a root-end filling material and was FDA-approved in 1997 (6). ProRoot MTA was the initially available commercialised MTA solution introduced in 1999 (6, 7). Since ProRoot MTA started out grey, all following versions have received upgrades on it. ProRoot MTA has intrinsic restrictions such as prolonged setting, high cost, and potential discolouration (8).

The last decade of the 2000s and the beginning of 2010s saw the development and use of additional bioceramics in endodontics. They exhibit physiological qualities like MTA, including antimicrobial properties, minimal toxicity to cells, and minimal inflammation (9, 10). The clinical setting uses Biodentine, ERRM, BioAggregate, Calcium-enriched Mixtures (CEM) on regular basis (11). As a “dentine substitute,” Biodentine became available in 2009 to enhance its ability to penetrate into dentinal tubules (12). Biodentine, made with conceptualisation of MTA production technique, has higher durability and more rapid setting as it incorporates neither calcium aluminate nor calcium sulfate (13).

Most of the studies on calcium silicate–based bioceramics have demonstrated favourable biocompatibility and sealability, emphasising their physicochemical properties rather than their regenerative healing potential. However, limited evidence exists regarding their ability to promote true apical closure and tissue regeneration, especially in comparison with biologically derived scaffolds. The apical healing capacity of traditional bioceramic cements and biologically produced scaffolds, like amniotic membrane, has not been compared in any research. Researching biologically active materials that can support the actual regeneration of tissues is necessary to close this gap. Therefore, this review aimed to assess the healing of immature teeth with biological scaffolds and compare their efficacy with conventional bioceramic materials.

MTA Angelus (Angelus, Londrina, Brazil) with gray and white formulations was introduced in 2001 and received the FDA approval in 2011. Bismuth oxide and Portland cement were incorporated into MTA Angelus. Due to the absence of gypsum, MTA Angelus had decreased setting time of less than 50 min when compared to ProRoot MTA (14).

Endo Sequence BC Putty (Putty form) and BC Sealer (Paste form) belong to Endo Sequence root repair material (ERRM). ERRM is a water-soluble 2CaO·SiO_2_ compound that creates hydroxyapatite after hardening (14). Excellent serviceability and reduced tooth discolouration risk characterise this group of prepared-to-use bioceramics (14). Ca₃(PO4)₂ and Silica are available in BioAggregate making it an Al-free bioceramic. BioAggregate has strong binding tenacity and securing qualities but low mechanical properties (15). Initially used in dentistry in 2008, CEM is a combination of several calcium-containing substances and has comparable characteristics to MTA at a lower cost. It has analogous both physical and therapeutic qualities as MTA but a distinct chemical constitution. Then the resin-formulated calcium silicate compound TheraCal LC hit the stores in 2011 as a subbase for direct and indirect pulp capping (16).

Over the past decade, bioceramic products have been widely investigated in endodontics. Some research evaluated the efficiency and therapeutic effects of currently available bioceramics, whereas others evaluated updated devices like Endo Sequence fast-set putty and BCS sealer HiFlow (17).

Latest materials like novel AGM MTA (AGM, Tehran, Iran) have been recently introduced into dentistry. This material is a calcium silicate-based material and is said to produce calcium ion release, with higher alkalinity & radiopacity without affecting the aesthetics of the tooth. This material is in the form of powder and liquid, with zirconium oxide as the opacifier. This material has been claimed to have excellent biocompatibility due to the presence of zirconium oxide. The thickening agent present in the liquid portion of AGM MTA has been shown to have better handling properties (18).

Another latest bioceramic material in endodontics is the development of premixed calcium silicate-based root canal sealer, AH Plus BC (Dentsply Sirona, USA) has been claimed to have better handling abilities when compared to resin based sealers. They are said to offer the advantages of a bioceramic formulation that increases alkalinity and releases calcium ions to promote the production of mineralised tissue (19).

**Figure 2:**
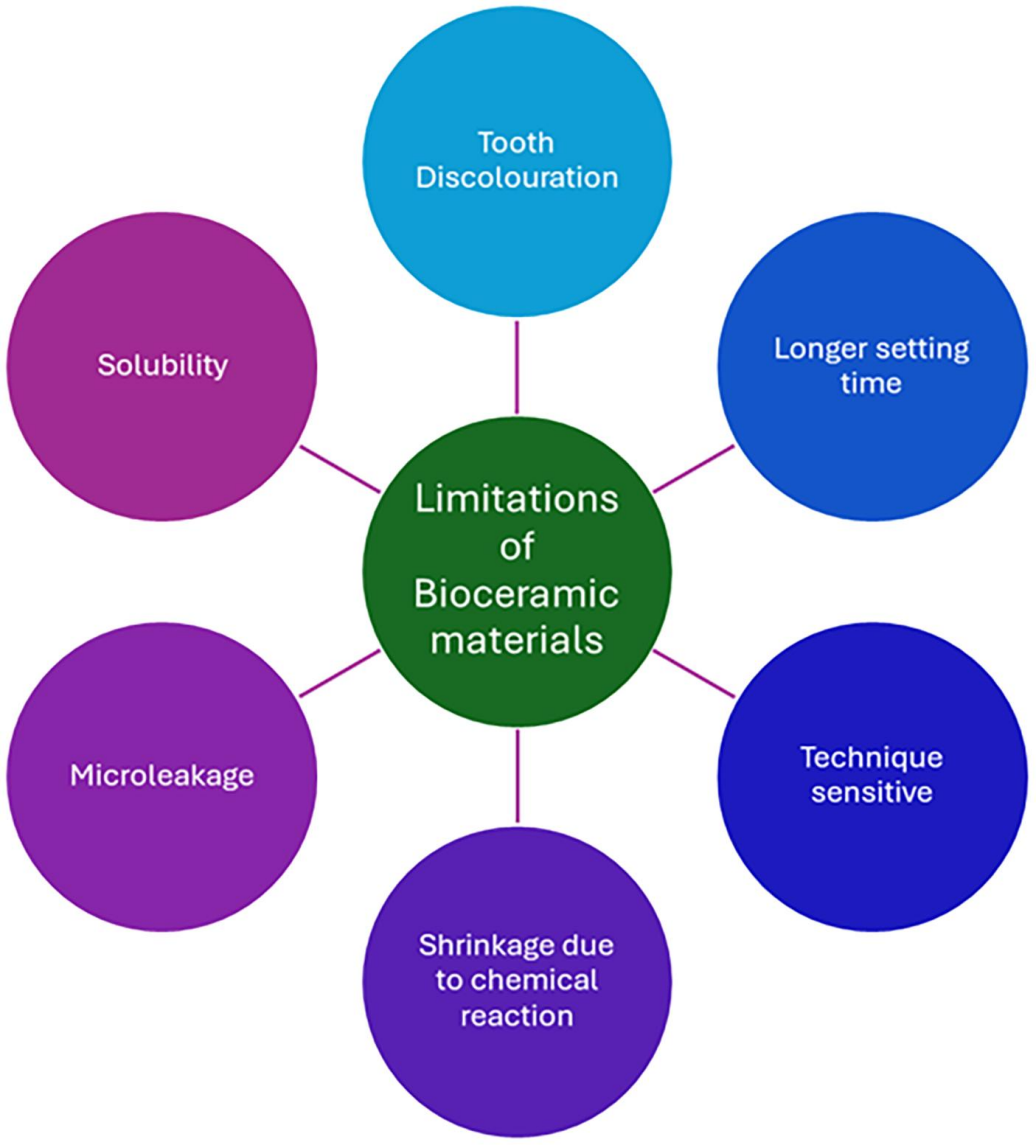
Limitations of various commercially available bioceramic materials.

Despite the recent advances in nanotechnology, there are no perfect bioceramic materials for endodontic applications to date, underscoring the necessity for ongoing innovation to address their inherent limits. Although they improve the qualities of endodontic materials, nanoparticles require expensive resources. Various nanoparticles like Ag, graphene, chitosan, etc, nano-polymers, and carbon-based nanoparticles like carbon nanotubes are all employed for endodontic applications. These nanoparticles must be carefully assessed for cytotoxicity and biocompatibility; nevertheless, they may present possible toxicity issues. Therefore, prospective clinical evidence will be required to evaluate these nanomaterials' efficacy and safety. However, various limitations of various bioceramic materials are listed in Figure 2 (20).

The invention of bioceramics has tremendously revolutionised endodontics. This article discusses bioceramics and their therapeutic uses in endodontics, such as retro-filling, pulp space therapy, deep caries management procedures, regenerative procedures, perforation and root defect repair. The limits along with prospective remedies for improving the application of bioceramics to endodontic treatment, are also discussed.

## 2 Search strategies

For last ten years, we searched *PubMed* and *Web of Science* search engines for all pertinent studies pertaining to this topic with all study designs. MeSH terms included Ceramics, Dental Cements, Biocompatible Materials, Bioceramics, and Endodontics. A manual search was conducted from highly reputed endodontic journals from the last few years. The chosen articles were referenced to find further papers. The main terms for bioceramics-related endodontic studies include retrograde filling, endodontic therapy, deep caries management, apexification, regenerative endodontic therapies, perforation repair, and root defect repair.

## 3 Properties of bioceramic materials

### 3.1 Chemical properties of bioceramic materials

To have a broad idea about the different bioceramic materials used in the endodontic therapy, Table 1 gives an insight about the composition of various materials (18–20). Bioceramic materials are available in different forms, namely, powder and liquid, putty form, and paste form. MTA, Biodentine, BioAggregate, and CEM belong to powder-liquid system. The powder component consists primarily of di, tri-calcium silicate, whereas the liquid component is water. Combining powder and liquid creates a moistened calcium silicate gel combo that hardens forming a rigid structure. BC Putty, a premixed bioceramic made of calcium silico-phosphate. TheraCal LC is a calcium silicate resin paste conjugated with type III Portland cement and set by light curing unit. EndoSeal MTA & BC Sealer belong to calcium silicate sealers in injectable form, where Al is present in EndoSeal MTA, and not in BC Sealer. MTA Fillapex, BioRoot RCS, Tech BioSealer etc are 2-paste sealers with active components of MTA, tri-Ca silicate, and CEM.

**Table 1:** Composition of various commercially available bioceramic products used in endodontics.

Material	Type	Composition	Manufacturer/Brand
Mineral Trioxide Aggregate (MTA)	Powder-liquid system	Calcium Silicate Cement & water	ProRoot MTA, MTA-Angelus
Biodentine	Powder-liquid system	Calcium Silicate Cement, water, calcium chloride, and hydro soluble polymer	Biodentine (Septodont)
Tech Biosealer	Powder-liquid system	Mixture of Calcium Silicates and Calcium Phosphates with Dulbecco's phosphate-buffered saline (DPBS)	Tech Biosealer (Profident)
Ceramicrete	Powder-liquid system	Mixture of Calcium Silicates and Calcium Phosphates with deionized water	Ceramicrete (Argonne National Lab)
iRoot BP	premixed bioceramic putty	Mixture of Calcium Silicates and Calcium Phosphates	iRoot BP (Innovative Bioceramix Inc.)
iRoot BP Plus	premixed bioceramic putty	Mixture of Calcium Silicates and Calcium Phosphates	iRoot BP Plus (Innovative Bioceramix Inc.)
iRoot FS	premixed bioceramic paste	Mixture of Calcium Silicates and Calcium Phosphates	iRoot FS (Innovative Bioceramix Inc.)
EndoSequence BC Sealer	premixed injectable	Mixture of Calcium Silicates and Calcium Phosphates	EndoSequence BC Sealer (Brasseler)
MTA Fillapex	Two-paste system	Calcium Silicate Sealer with Salicylate resin, bismuth trioxide, and fumed silica, Fumed silica, titanium dioxide, MTA (40%), and base resin	Angelus
BioRoot RCS	Powder-liquid system	Calcium Silicate Sealer with Water, calcium chloride, and hydrosoluble polymer	BioRoot RCS (Septodont)

### 3.2 Biocompatibility of bioceramic compounds

The biological acceptability and biological function of bioceramics mostly depend on how they relate with tissue surrounding them. Bio Ceramics impact stem cell division, multipilcation, differentiation, emigration, cell death, and immunologic cell activity. Cellular reaction to bioceramics impacts wound repair and tissue restoration (21). Dental tissue-derived mesenchymal stem cells (MSCs) include Dental Pulp Stem Cells (DPSCs), Human Exfoliated Deciduous Teeth Stem Cells (SHED), and apical Stem Cells from the Apical Papilla (SCAPs) (22). MSCs' regeneration and multifaceted differentiation capacity are crucial for pulp rejuvenation and bone formation (23). Bioceramics enhance stem cell adhesion and their survival, with effects varying by cell type (24).

Biodentine, NeoMTA Plus, and TheraCal LC exhibit good tissue adaptability and lead to odontogenesis or osteogenesis due to the proliferation of MSCs (25). Both ProRoot MTA and Biodentine exhibit biological features that support DPSC functioning ex-vivo (26). Biodentine promotes dentinogenesis via Mitogen-Activated Protein Kinase (MAPK) and CaMKII pathways (27). ProRoot MTA, Biodentine, and ERRM may promote SCAP mineralisation and odontogenesis, thereby enabling pulp regeneration (28).

Osseous repair depends on the number of osteoblasts and osteoclasts around the damaged tissue. When bioceramic materials are used in cases of perforation repair, and retro-filling, the work between the material and the cells is very important for the reduction of infection and wound recuperation (29). MTA has shown to effectively reduce bone resorption by impeding Receptor Activator of Nuclear factor Kappa-B Ligand (RANKL)-mediated osteoclast activity and its production (30). BioAggregate drives formation of osteoblasts, inhibiting osteoclastogenesis, and significantly reducing osseous resorption *in-vivo* (31–33).

DPCs/PDLCs aid in healing wounds and bring about tooth-tissue regeneration. Bioceramics react to DPCs/PDLCs for deep caries management procedures, perforation repair, and retro-filling. MTA, Biodentine, BioAggregate, and ERRM increase DPCs' transcription of genes involved in mineralisation and odontoblastic maturation (34–36).

Much research have examined the biocompatibility and bioactivity of bioceramics in endodontics. MTA is the most researched material and regarded the “benchmark”. Limited research exists comparing bioceramics to MTA, and *in vitro* models have varying approaches and outcomes. Additional trials are required to offer persuasive proof for using such materials in endodontic procedures.

## 4 Applications of various bioceramics in endodontics

**Figure 3:**
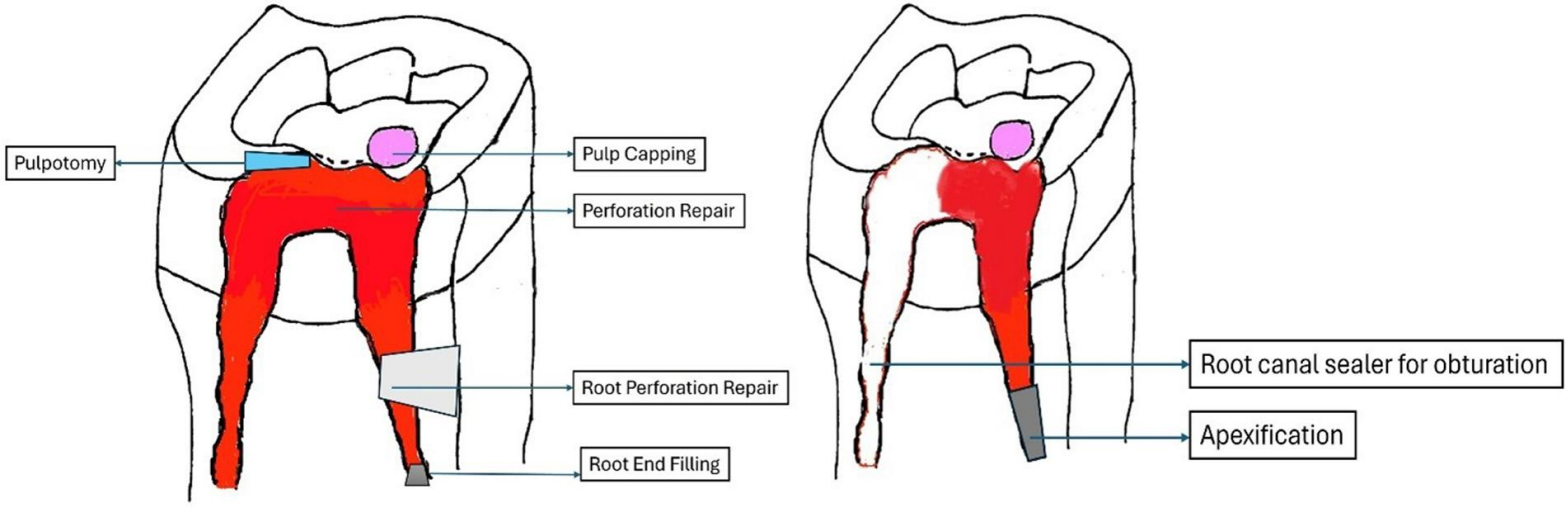
Schematic representation of clinical application of bioceramics.

Lately, endodontic procedures have started employing bioceramics extensively (Figure 3). Materials like MTA, Biodentine, BioAggregate, BC Putty, and CEM are frequently employed for retrograde obturation, Deep caries management procedures, apexification/regenerative endodontic therapy, repair of perforation sites, and repair of root defects. Bioceramics in the paste form like BioRoot RCS and BC Sealer are routinely used in enddodontic obturations.

### 4.1 Apical end filling

The apical end of the pulp chamber can be filled using orthograde or retrograde manner to achieve apical sealing. A suitable apical closure product ought to be bioactive, biocompatible, long-lasting, function well, and stimulate tissue regeneration (37, 38). Several dental restoration materials, including bioceramics like MTA, are commonly utilised as root-end fillings in dentistry (39).

**Figure 4:**
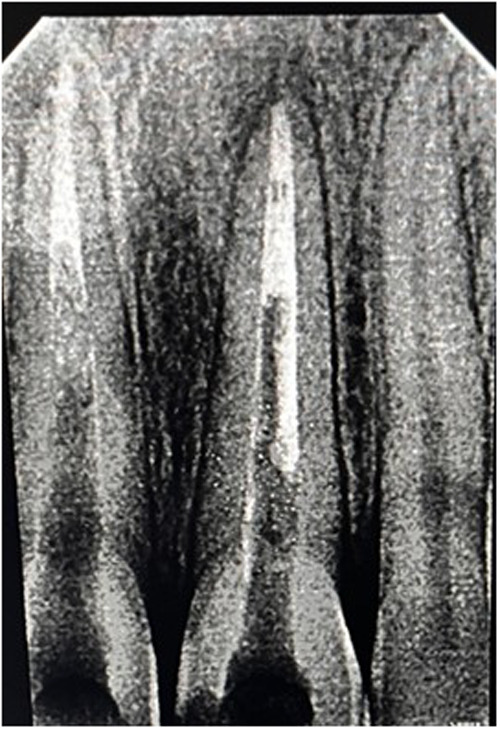
IOPA radiographic representation of orthograde obturation of pulp space with MTA.

**Figure 5:**
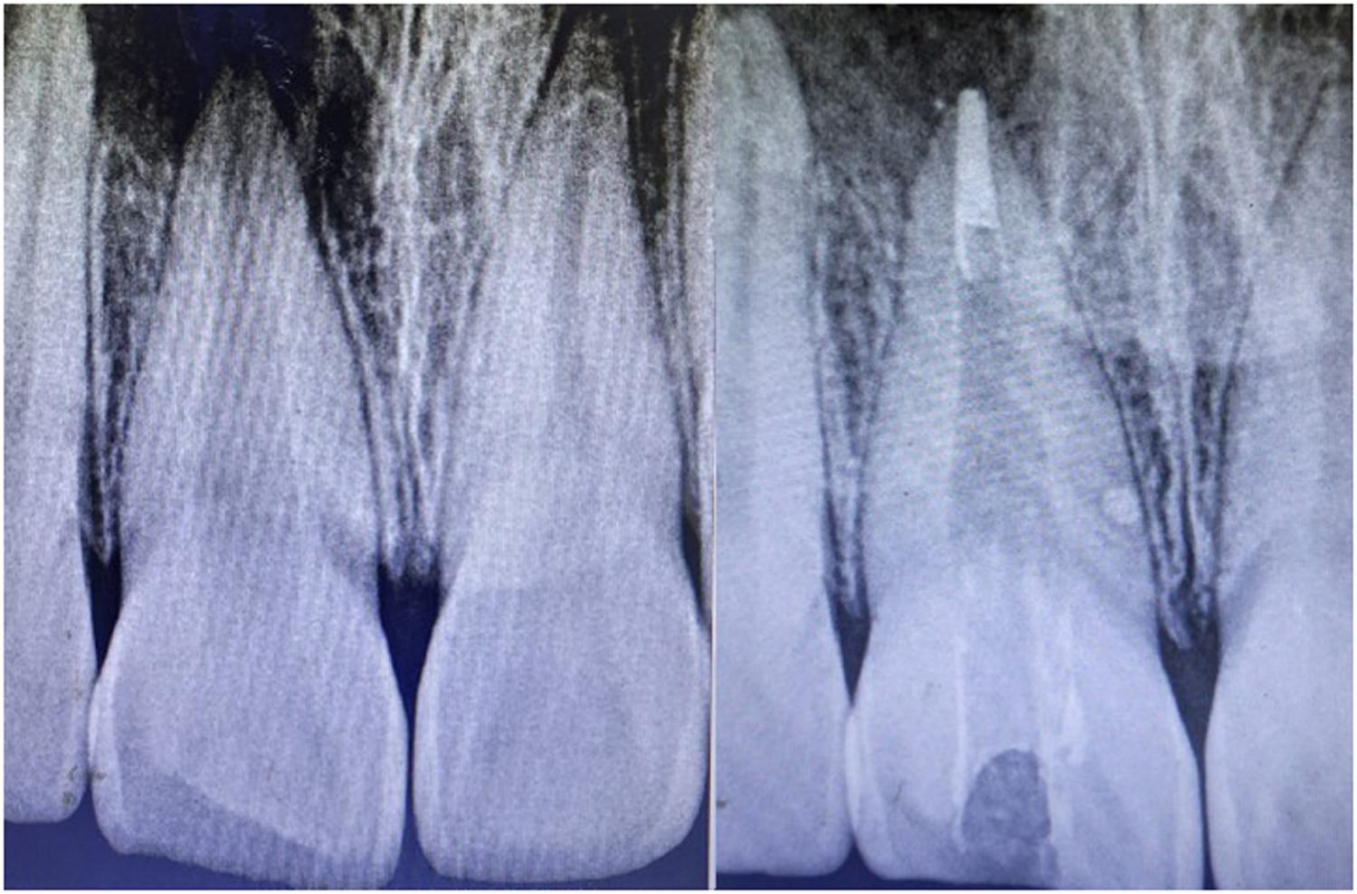
IOPA radiographic representation of biodentine apical plug for a tooth with open apex.

Orthograde obturation of the pulp space refers to the sealing of the apical end of the pulp space by carrying bioceramic materials like MTA, Biodentine, etc from the coronal end to the radicular end of the pulp space to form a apical plug (40) (Figure 4). MTA has become extensively utilised in the apical barrier approach with for a propsective clinical and radiological results (41–43). A lot of case reports have been published with various bioceramic materials being applied as apical barrier and has provided good results (44–46). Apical plugs (Figure 5) in immature permanent teeth with MTA, Biodenitin, CEM, etc have shown to have high fracture resistance (47, 48). Taken into account that MTA is the most frequently suggested material for apical barriers, while Biodentine, BioAggregate, and CEM need further excellent research to confirm its efficacy in this clinical context.

### 4.2 Endodontic microsurgery

**Figure 6:**
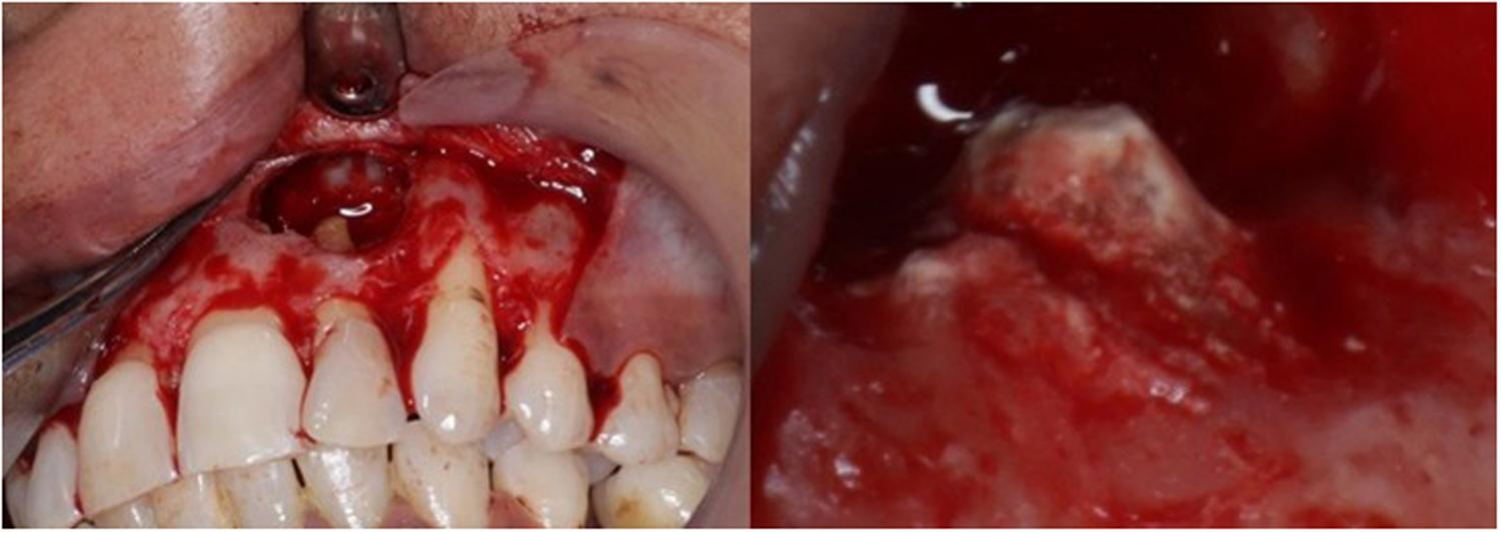
Clinical presentation of application of biodentine for root end filling during endodontic microsurgery.

Apical surgery is useful for teeth retention in severe periapical disorders. The clinical results of apical surgery depend on meticulous root-end filling, which is essential to preventing microleakage and recurrence of infection (49). Bioceramics like MTA are frequently utilised in apical surgery due to their biological suitability, superior sealing, infectious microorganism suppression, and capacity to stimulate periapical tissue repair (50). Retro-filling with bioceramics (Figure 6) is used for preventing the recurrent infection of periapical lesions in a surgical approach (51). Retrograde filling is instituted after 3 mm of apical abscission with 3 mm of apical retro-preparation which constitutes as the most important step in microsurgery and replantation cases (52, 53). In difficult clinical settings that necessitate quick material setting, fast-setting CSCs in EMS are advised (54). Twelve months effectiveness rates of MTA and BC Putty all scored above 93% in randomised clinical trials, demonstrating a good outlook (55, 56).

### 4.3 Pulp space therapy

**Figure 7:**
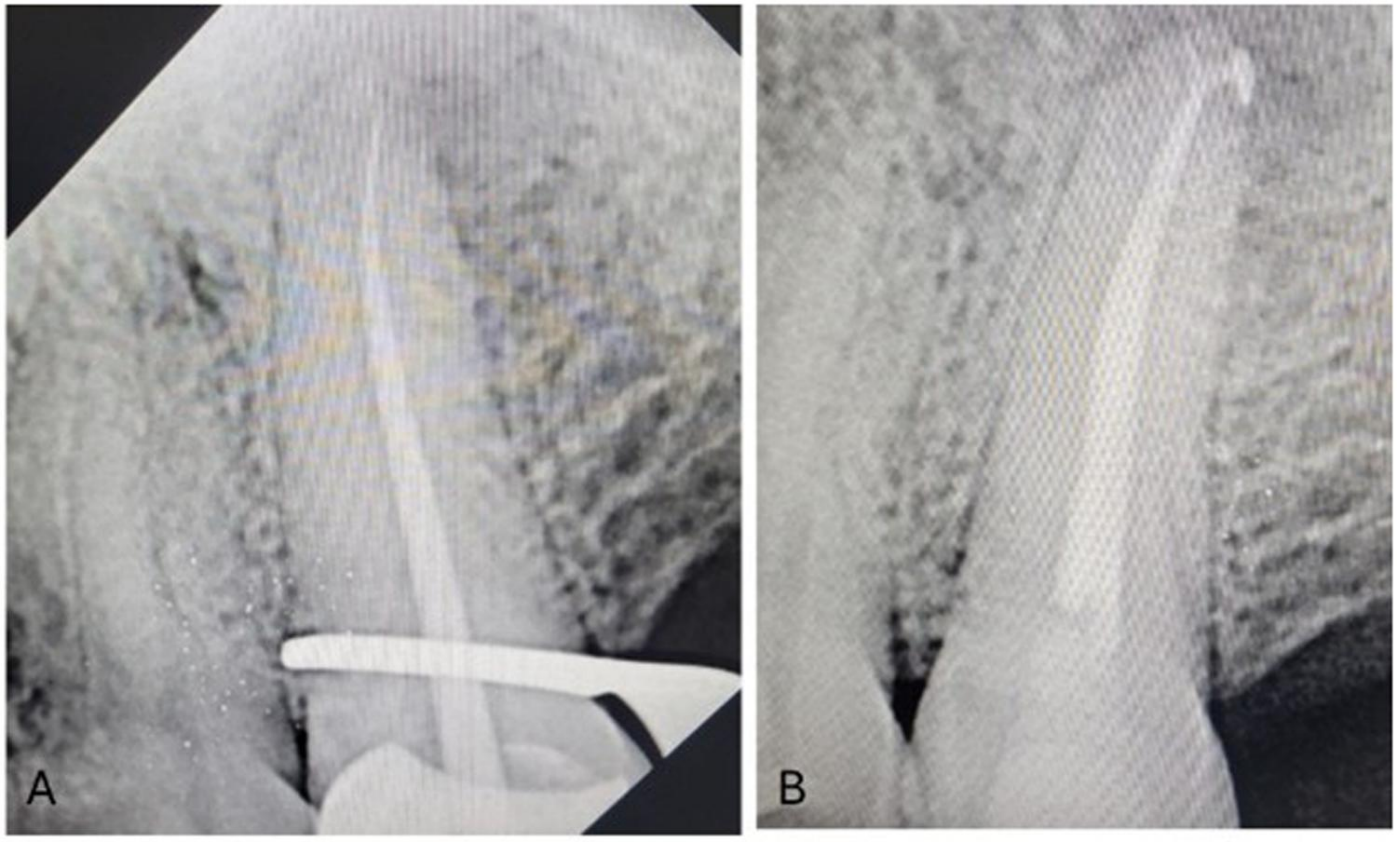
**(A)** Preoperative IOPA radiographic representation of pulp space therapy with the use of bioceramic sealer. **(B)** Postoperative IOPA radiographic representation of pulp space therapy with the use of bioceramic sealer.

Pulp space therapy is the considered to be the most successful and prevalent treatment for pulpo-periapical diseases. Single sitting with single cone endodontic therapy is time and cost effective and better patient compliance (57). The Gentle Wave system uses enhanced fluid motion to minimise conventional preparation's overuse and reduce the possibility of Ni–Ti rotary instrument canal separation (58–60). Pulp space obturation, specifically for irregular pulp spaces, is done with hydraulic obturating material and bioceramic sealer (Figure 7). These methods progressively rely upon the root canal sealer, therefore its ability to flow and other biophysical qualities are critical to the effectiveness of the treatment (61). Bioceramic sealers like BCSealer are biocompatible, adaptable, and chemically stable. After applying the single-cone approach, bioceramic sealers have shown good immediate clinical effects (62, 63). Endodontic obturation with gutta-percha/bioceramic sealer has a reduced postoperative pain and better recovery than standard sealer (64).

According to current research, the single-cone approach using bioceramics provides good patient outcomes and operational reliability. The majority of practitioners don't embrace it since there are no standardised clinical criteria and they are dependent on root canal sealers. It's contentious to employ the single-cone approach, and it calls for large-scale clinical trials.

### 4.4 Deep caries management treatment strategies

**Figure 8:**
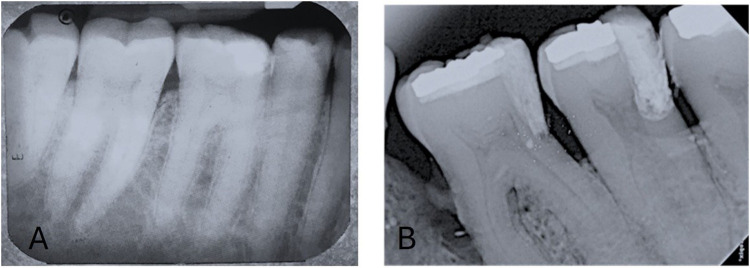
Postop IOPA radiographic representation of vital pulp therapy with the use of bioceramic materials. **(A)** Indirect pulp capping with Biodentine and, **(B)** Direct Pulp capping with MTA.

Recently developed bioactive restorative materials have led to more conservatively and minimally invasive procedures for exposed dental pulp (65, 66). Vital Pulp Therapy modalities like pulp capping (direct and indirect) and pulpotomy are conservative approaches for maintaining the dental pulp vitality and functioning of pulp after injury (67, 68). Choosing the right capping material is crucial, and MTA is frequently employed and investigated. AAE consensus recommends CSCs in deep caries management due to their constant clinical performance (69) (Figure 8).

**Figure 9:**
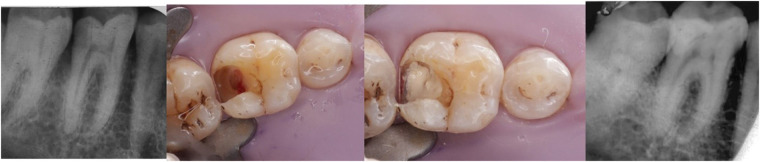
Clinical procedure of direct pulp capping with biodentine.

In dental restorations, pulp capping whether direct or indirect is done to protect the dental pulp when it has been subjected or almost exposed during tooth preparation, from iatrogenic exposure, or from a deep carious lesion that has breached (65, 70). Most research has focused on MTA in pulp capping. MTA utilised in VPT achieves consistent therapeutic results and maintains the functionality of pulp better than calcium hydroxide (71–73). BioAggregate is a viable pulp capping substitute to MTA and offers great invitro biocompatibility (74). BC Putty can produce reparative dentinal bridges and is biocompatible for pulp tissue (75, 76). The clinic-histological effectiveness of biodentine in VPT is superior to that of calcium hydroxide, as shown by decreased postoperative discomfort and sensitivity, stronger dentinal bridge development, and decreased pulpal infection (65, 77, 78). Contingent on the current literature available, bioceramic materials produce definitive dentinal bridge and maintain the dental pulp functionality and integrity. As per literature, MTA and Biodentine are the most studied materials for pulp capping and has shown to have excellent results (Figure 9).

**Figure 10:**
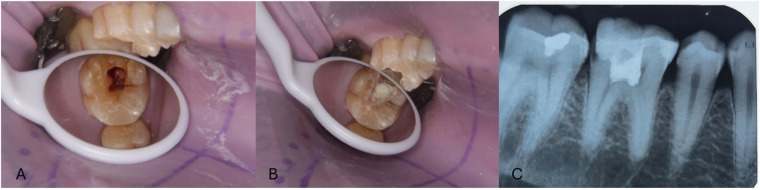
Clinical and postop IOPA radiographic representation of pulpotomy with the use of biodentine. **(A)** Full pulpotomy and, **(B)** Application of Biodentine at pulpotomy site, **(C)** Post IOPA Radiograph following Pulpotomy procedure.

Pulpotomy is a procedure to remove inflamed coronal pulp and restore the radicular pulp to maintain health (79). Pulpotomy can be complete or partial depending on the extent of the dental pulp is resected (80). MTA in pulpotomy (Figure 10) can yield excellent effects, backed up by superior proof (81–83). But in recent times, pulpotomy with Biodentine has shown to have good saleability and superior results (84–86). BC Putty has shown to have excellent patient compliance to partial pulpotomy and can be considered as one of the options (86, 87).

### 4.5 Regenerative endodontic procedures

Dental stem cells are proven to induce root formation and development in immature young permanent teeth, hence apexification and regenerative endodontic procedures are considered as viable options of tissue healing and apical end closure (88, 89). Combination of regenerative procedures and biomaterials has shown successful clinical results (90).

**Figure 11:**
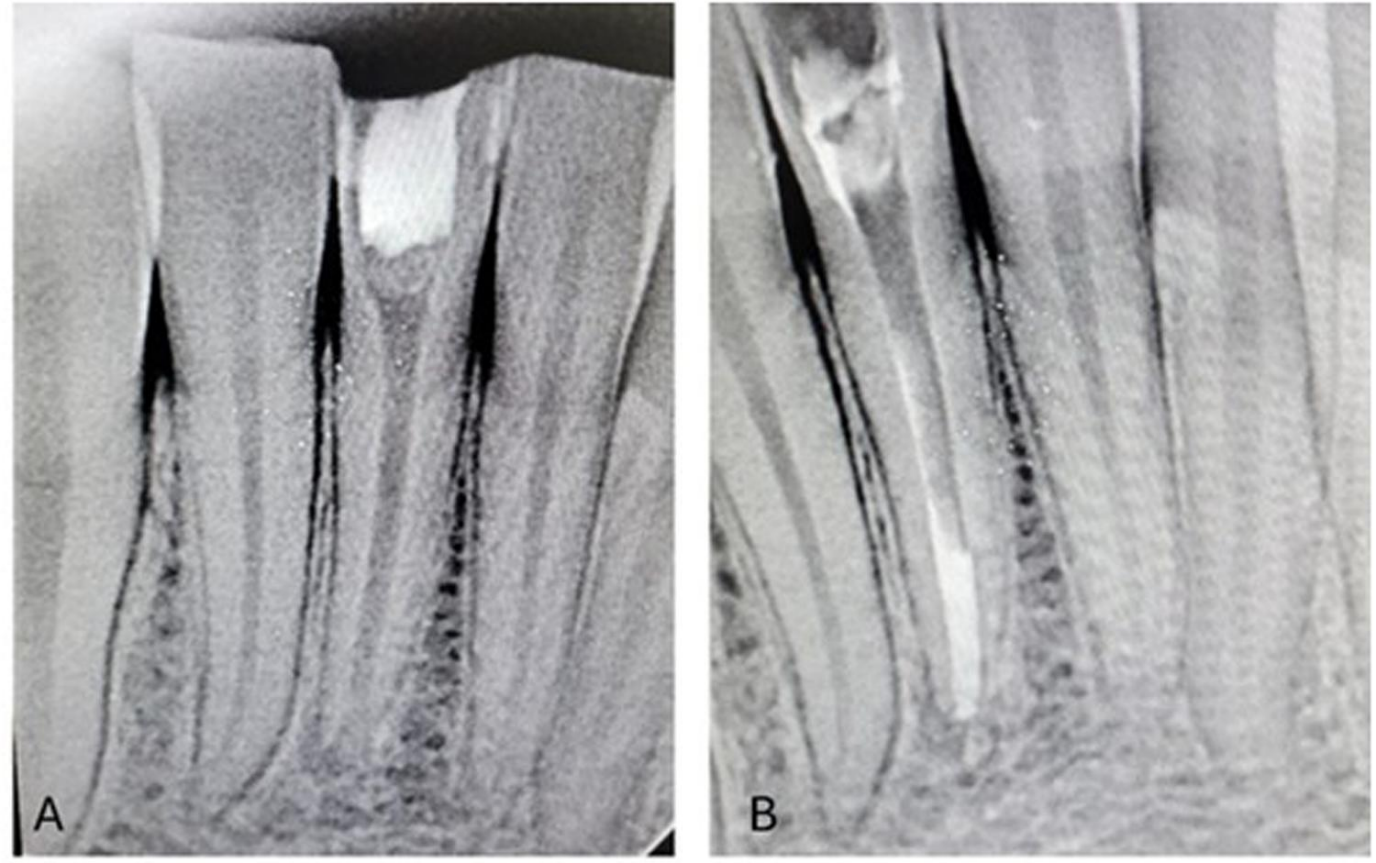
Postop IOPA radiographic representation of regenerative endodontic procedures with the use of biodentine. **(A)** Placement of amniotic membrane, **(B)** Application of Biodentine as apical plug.

Apexification is termed as the root end closure in the immature young permanent teeth with the introduction of bioactive materials (91). Apexification with MTA (Figure 11) results in improved therapeutic closure, less inflammation, and reduced intervention periodicity and tooth fracture risk (92–94). Newer biocermamic materials have shown to superior results (95). Biodentine and ProRoot MTA effectively reduce premature fractures of the root in within the initial 30 days of apexification, surpassing Neo MTA Plus (96). Biodentine apexification may boost fracture resistance of young permanent roots, according to several studies (97–99). In addition, BC Putty helps young teeth with nonvital pulp in root formation and maturation (100).

Regenerative Endodontic Therapy (RET) is considered as alternative option to apexification in some cases and shown to have increased root thickness and length (101). Revascularisation, or blood coagulation, is a prevalent RET technique that induces coagulation of blood in the periapical tissues of teeth after removal of the infection. This process employs stem cells from around the root to encourage proliferation, differentiation, and development of “new pulp tissues” in the root canal (102, 103). MTA is probably the most used capping material in RET, with a high overall longevity rate (104). The capping material for revascularization is made to come in intimate contact with coagulated blood, and hence it should be bioregenerative, bioactive, biocompatible and antimicrobial (105). Improved bioceramics are promising for coronal capping of previously placed blood coagulation stents. Biodentine, ProRoot MTA, etc have shown to promote the differentiation of SCAP, which aid in the formation of root development considering as one of the best options for RET (106). The accidental release of TGF-β 1 from pulp space dentin by biodentine led to increased mineralisation of human apical papilla cells compared to Pro-RootMTA (107). Many case studies have shown that Biodentine when employed as RET barrier produced favourable outcomes (23, 108). RET is an envisaged approach for pulp necrosis in juvenile teeth with bioceramics and more top-notch investigations are needed.

### 4.6 Repair of perforation sites

**Figure 12:**
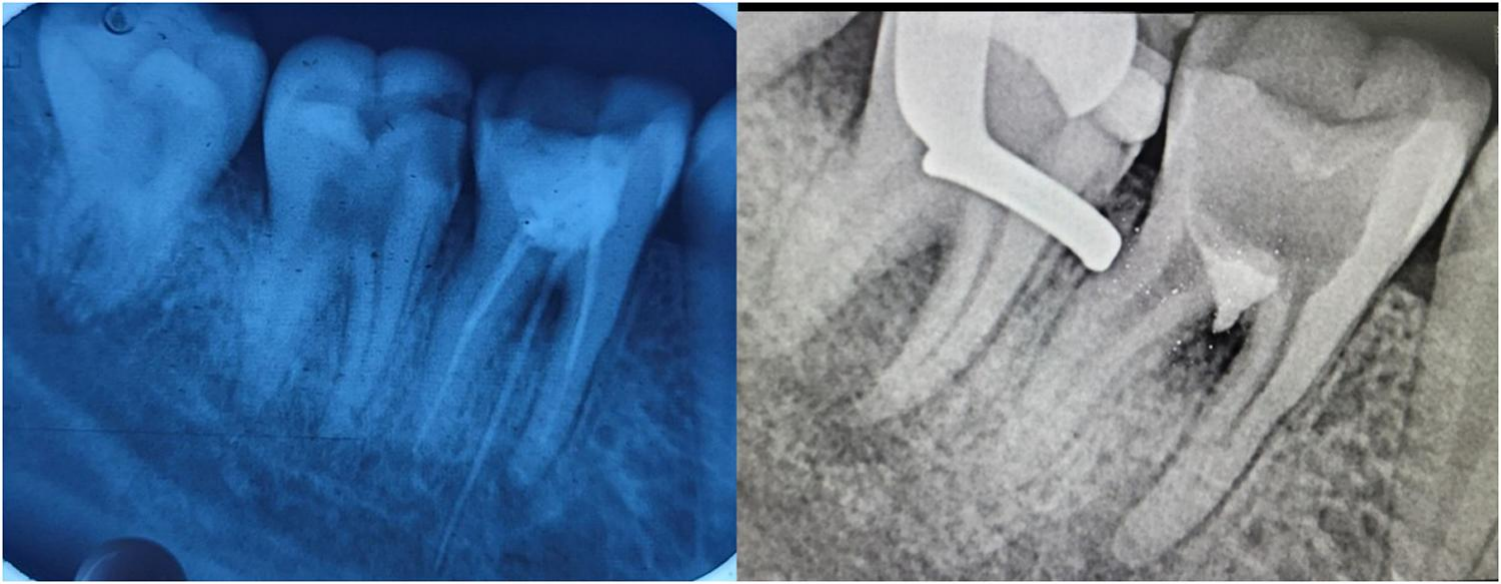
Preop & postop IOPA radiographic representation of perforation repair with the use of biodentine.

Tooth perforation is connection between pulp space with periodontal space (109). Perforation repair by biotic biodegradable cements is an important point for clinical intervention. Repair of the perforation sites are mostly iatrogenic in nature. Ca (OH)_2_, MTA, and CSCs are among the frequently suggested root perforation sealers (110). The preferred material for perforation repairs (furcation and root) is MTA, which can induce a beneficial histological reaction (111). Neo MTA Plus has comparable saleability and better biocompatibility to MTA Angelus, Endo Seal, and ProRoot MTA (112, 113). Biodentine (Figure 12) and MTA have shown to cause similar periradicular inflammation and bone resorption when employed for sealing perforation sites (114). Biodentine and MTA minimise tension on the perforation sites that could be hazardous (115). Blended bioceramics are potential alternatives for mending deciduous molar perforations, with superior seal and therapeutic results than MTA (116).

Many invasive and noninvasive procedures are utilised to treat root abnormalities like the palate–radicular groove and root resorption, which have a bad prognosis (117). Bioceramics are highly recommended as they can interact with periapical and periodontal tissues promoting genesis of surrounding tissues (118). Palatal-Radicular Groove is a genetic disorder, and must be restored to prevent the spread of infection after biomechanical preparation with materials such as GIC, flowable resins, and CSCs (119). In addition, Biodentine is being utilised to seal PRG to preserve damaged teeth with mixed periodontal diseases (120–122).

### 4.7 Repair of root resorption (internal & external)

**Figure 13:**
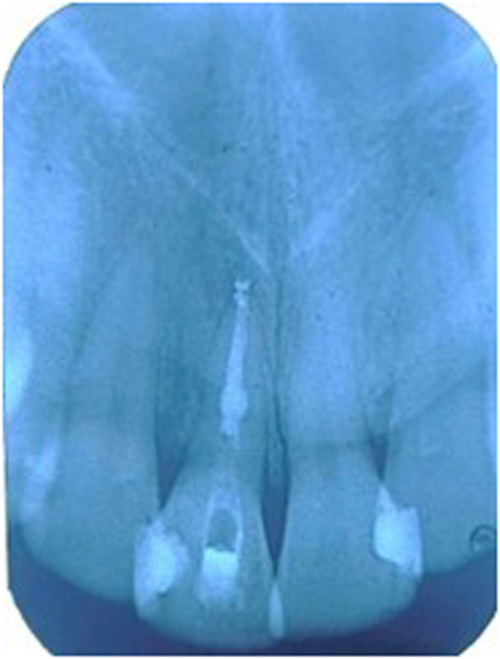
IOPA radiographic representation of obturation of internal resorption using bioceramic sealer and thermoplasticised gutta percha.

Root resorption (internal &external) refers to the loss of dentinal tissues on the internal and external surfaces (123). The spot, level, and amount of root resorption determine whether it is treated conservatively or surgically (124). Root resorption and perforation often occur concurrently, and MTA (Figure 13) has recently been shown to have good long-lasting effects (125–127). Newer bioceramics have also been studied. Filling the interior reservoir of resorption site with BC Putty, MTA, and Biodentine provides better fracture toughness than gutta-percha/sealer (128). Bioceramic putty is an efficient nonsurgical remedy for external cervical resorption (129). Bioceramic sealers are alkaline, sustained calcium release, and root-reinforcing capability and can cure root resorptive defects with a favourable outcome (130).

## 5 Outlook

Till date, MTA is the most researched bioceramic material in Endodontics. MTA is the benchmark for developing innovative bioceramics and offers a consistent clinical outcome in endodontic therapy. Each bioceramic material has limits in practical uses and cannot be considered perfect. The evolution of newer materials has led to the creation of bioceramics beyond MTA, including Biodentine, BioAggregate, BioRoot RCS, etc. Recently developed materials are utilised in retrograde filling, pulp space therapy, deep caries management strategies, regenerative endodontic procedures, repair of resorptive and perforation sites. They have demonstrated similar or better therapeutic results than MTA in *in-vivo* studies, ex-vivo investigations, and case reports. The availability of superior clinical research with continual monitoring and controlled effectively laboratory studies is limited. Further research of outstanding quality is necessary to confidently use bioceramics in endodontics. Bioceramics are crucial for treating endodontic disorders and have promising future possibilities. Researchers anticipate future development of new or superior bioceramics.
